# Optimal distribution of piezoelectric patches for active vibration reduction of a thick plate using singular value decomposition approach

**DOI:** 10.1038/s41598-021-93136-5

**Published:** 2021-07-02

**Authors:** Azin Nadi, Mojtaba Mahzoon, Ehsan Azadi Yazdi

**Affiliations:** School of Mechanical Engineering, Shiraz University, 7193616548 Shiraz, Fars Iran

**Keywords:** Engineering, Materials science, Mathematics and computing

## Abstract

This paper uses the singular value decomposition approach to find the optimal distribution of a set of piezoelectric actuators and sensors in order to suppress the vibrations of a thick plate. The dynamic model of the system is derived using Mindlin plate theory and consequently, the finite difference method is employed to divide the thick plate to a finite number of nodes with appropriate horizontal and vertical distances. To compute the control force of piezoelectric actuators, the singular value decomposition approach for the column control matrix is supposed as the fitness function of an optimization problem. Through a genetic algorithm, the optimized solution is obtained. The results of numerical simulations indicate the optimal location achieved by the proposed method outperforms the previous results in suppressing the vibrations of a thick plate.

## Introduction

The problem of finding the optimal configuration of piezoelectric patches on a flexible plate for vibration suppression is one of the primary interests in engineering design. In smart structures, sensors and actuators work in a control system to achieve a higher performance in vibration suspension compared to the conventional passive systems. In such systems, piezoelectric sensors and actuators are widely used because they are inexpensive, reliable, fast, low weight, and low power^1^. Therefore, the optimal location of piezoelectric patches to improve controller optimality is one of the important open research problems. It is well known that misplaced patches (sensors and actuators) may lead to issues such as the lack of observability and controllability^2^.

Several studies have been conducted on the optimal position of piezoelectric actuators and sensors on thin plates by considering various objective functions and techniques. In terms of the objective function based on controllability and observability index, the optimal location of piezoelectric actuators on a composite structure by Padoin et al.^3^ and a thin plate using the Modified Control Matrix and Singular Value Decomposition (MCSVD) technique by Chhabra et al.^1^ was suggested. Moreover, the optimal position and the number of active elements for separate sensors and actuators were found by Bruant et al.^4^ and Chakraborty et al.^5^. Biglar et al.^6^ suggested an objective function based on the spatial controllability or observability Gramian and the incorporation of residual modes to decrease the spillover effect for the active vibration control of a plate to obtain optimal orientations and positions of piezoelectric patches. The optimal location of piezoelectric patches has been optimized with the objective function based on the maximization of Gramian matrix visibility and distributing the amount of strain energy on the plate by Loghmani et al.^7^, Ferrari and Amabili^8^, respectively. In addition, the H∞ norm of the closed-loop system as the objective function was considered in^9^. Active vibration suppression of a flexible structure bonded with optimized piezoelectric pairs was developed by Daraji and Hale^10^, and reduction of the genetic algorithm search space by designing a conditional filter was proposed in^11^. In other researches, the optimal location of sensors in structural health monitoring systems was studied to cover a specific number of low-frequency modes^12^. In large-scale structures due to moving loads, an approach based on convex relaxation was proposed by Błachowski et al.^13^ to efficiently optimize sensor layout. Also, the optimal places of triaxial accelerometers were presented based on Effective Independence by Kammer and Tinker^14^.

Plates are widely used as structural elements in modern construction aerospace, engineering, and aeronautical industries. Several models for the plates based on various theories were studied in the literature. The deflection of the Kirchhoff plate was first approximated with deep physical informed feedforward neural networks by minimizing a loss function related to avoiding a FEM discretization entirely in^15^. The proposed deep collocation and energy methods with a set of DNNs approximating the transversal deflection are proven to be effective in the bending analysis of the Kirchhoff plate^16^. The Mindlin plate theory is used to obtain the dynamic model of a thick plate^17^. Moreover, a three-dimensional piezoelectric structure was extended to the detection of inclusions by Nanthakumar et al.^18^ for inverse problems.

On the one hand, the majority of studies in the area of the active vibration suppression of plates and shells by optimal location of piezoelectric patches (actuators or sensors) have selected the number of patches arbitrarily and optimized just their location on a thin plate. However, this causes weak vibration suppression or high added weight, costs and energy requirement. Moreover, although many researches have been done regarding the active vibration control of smart structures, there are still scopes and needs to be improved. Moreover, the problem of optimal configuration of piezoelectric patches on flexible a structure where improved performance and efficiency is one of the most interesting issues among engineering design.

If the effects of transverse shear deformation do not consider in relatively thick plates, it causes error in the calculation of deflections and stress resultants of such plates. Because, thick plates are main members of various structures. They can be applied as elements of engine foundations, reinforced concrete bridges, parts of various floating structures^19,20^. In addition, it is uneconomical to implement the cost of sensors and actuators into account to mount patches on every part of a structure. Moreover, the weight is a vital factor in various industry especially aeronautical. The resulting configuration and number of sensors and actuators must be optimal. A contribution of the present paper is related to find a new objective function based on the optimum number of patches as well as their optimal location on the thick plate for vibration reduction by using a binary-coded genetic algorithm that modified by adding a feasibility test in the mutation step.

In this study, a finite difference model of a thick plate with piezoelectric sensors and actuators has been developed, and Linear Quadratic Regulator (LQR) optimal control scheme has been used to study the effectiveness of the controller. The results of the present research have raised the closed-loop average dB gain as compared to the optimal position of random selection, the norm of singular value decomposition, and using optimal placement which is obtained on a thin plate with the same number of patches.

The method has been used in the present study has the following advantages compared to the previous articles:

1. Instead of using the thin plate in^21^ for finding the optimal location of piezoelectric patches, in this paper, the optimization of piezoelectric patches locations on a cantilever thick plate based on Mindlin plate theory has been carried out.
2. Instead of selecting a specified number of piezoelectric patches on the thin plate in^1^, in this paper, the optimization of the number of piezoelectric patches on a cantilever thick plate has been performed.
3. Although the method presents in this paper for obtaining the fitness function somewhat similar to the method presented in^1^, this paper considers the optimal number of piezoelectric patches by using the Mindlin plate theory for modeling the cantilever thick plate. Moreover, the objective is to attenuate the vibrations by considering the modified control matrix and singular value decomposition and developing a cost function using a binary-coded genetic algorithm.

The paper is arranged as follow:

In "Thick plate formulation" section to "State-space model" section, the equation of motion of the thick plate with piezoelectric patches are solved through the FDM by applying the Mindlin plate theory and the LQR optimal control theory. In "Singular value decomposition (SVD)" section, Singular Value Decomposition has been discussed for finding a fitness function which is used in genetic algorithm. Then, we discuss the 1-bit coding of the piezoelectric actuators and sensors installed on the thick plate. The binary-coded genetic algorithm (GA) is employed to find the optimal placement and number of the patches on the cantilever thick plate in "1-bit coding plate by genetic algorithm" section. Moreover, average closed-loop dB gain reduction has been applied to validate the optimal placements of piezoelectric actuators and sensors obtained in "Average closed-loop dB gain reduction" section. Finally, the results and conclusion are presented in "Results and discussion" and "Conclusions" sections, respectively.

## Formulation and methodology

### Thick plate formulation

**Figure 1:**
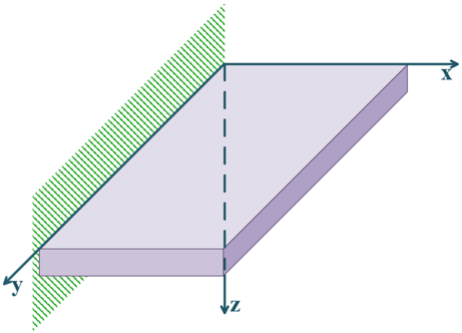
A cantilever thick plate.

Figure 1 shows the coordinate axes and boundary conditions of a thick plate.

The equation of motion for a rectangular Mindlin plate under time-varying dynamic load as a function $P\left( {x,y,t} \right)$ is (see Supplementary Note for detailed derivations):

1 $$\left[ {D\nabla ^{4} w_{b} - J\left( {1 + \frac{{\bar{m}D}}{{JS}}} \right)\frac{{\partial ^{2} }}{{\partial t^{2} }}\nabla ^{2} w_{b} + \bar{m}\frac{{\partial ^{2} }}{{\partial t^{2} }}\left( {w_{b} + \frac{J}{S}\frac{{\partial ^{2} w_{b} }}{{\partial t^{2} }}} \right)} \right] = P\left( {x,y,t} \right)$$

where $w_{b}$ is bending displacement of thick plate. $\bar{m} = \rho h$ is the mass per the unit area, $\rho$ and $h$ are density of the plate and plate thickness, respectively. $J = \rho I = \rho \left( {\frac{{h^{3} }}{{12}}} \right)$ is mass inertia moment of plate cross-section. $D = \frac{{Eh^{3} }}{{12\left( {1 - \upsilon ^{2} } \right)}}$ and $S = kGh$ are the bending and shear strength of the plate, respectively. $E,G,\upsilon ,k$ are Young's modulus, Shear modulus, Poisson's ratio, shear correction coefficient, respectively. $\nabla ^{2} \left( \cdot \right) = \frac{{\partial ^{2} \left( \cdot \right)}}{{\partial x^{2} }} + \frac{{\partial ^{2} \left( \cdot \right)}}{{\partial y^{2} }}$ is the Laplace differential operator. $\nabla ^{4}$ is a differential operator equivalent to $\frac{{\partial ^{4} }}{{\partial x^{4} }} + 2\frac{{\partial ^{4} }}{{\partial x^{2} \partial y^{2} }} + \frac{{\partial ^{4} }}{{\partial y^{4} }}$.

Equation (1) can be rewritten based on internal moments.

2 $$\left[ {\frac{{\partial ^{2} M_{x} }}{{\partial x^{2} }} + 2\frac{{\partial ^{2} M_{{xy}} }}{{\partial x\partial y}} + \frac{{\partial ^{2} M_{y} }}{{\partial y^{2} }} - \frac{J}{{D\left( {1 + \upsilon } \right)}}\left( {1 + \frac{{\bar{m}D}}{{JS}}} \right)\frac{{\partial ^{2} }}{{\partial t^{2} }}\left( {M_{x} + M_{y} } \right) - \bar{m}\frac{{\partial ^{2} w_{b} }}{{\partial t^{2} }} - \frac{{\bar{m}J}}{S}\frac{{\partial ^{4} w_{b} }}{{\partial t^{4} }}} \right] = - P\left( {x,y,t} \right)$$

$M_{x} ,M_{y}$ and $M_{{xy}}$ are the internal moments of the thick plate.

If N piezoelectric actuators are fully connected to the thick plate. The patches will generate moments which effect on the internal moments of the thick plate. Because piezoelectric patches are usually lighter and smaller than the host plate, the effect of their mass on dynamics of the plate can be ignored. Therefore, the above equations are modified as follows:

3 $$\left[ {\frac{{\partial ^{2} \left( {M_{x} - m_{{px}} } \right)}}{{\partial x^{2} }} + 2\frac{{\partial ^{2} \left( {M_{{xy}} - m_{{pxy}} } \right)}}{{\partial x\partial y}} + \frac{{\partial ^{2} \left( {M_{y} - m_{{py}} } \right)}}{{\partial y^{2} }} - \frac{J}{{D\left( {1 + \upsilon } \right)}}\left( {1 + \frac{{\bar{m}D}}{{JS}}} \right)\frac{{\partial ^{2} }}{{\partial t^{2} }}\left( {M_{x} - m_{{px}} + M_{y} - m_{{py}} } \right) - \bar{m}\frac{{\partial ^{2} w_{b} }}{{\partial t^{2} }} - \frac{{\bar{m}J}}{S}\frac{{\partial ^{4} w_{b} }}{{\partial t^{4} }}} \right] = - P\left( {x,y,t} \right)$$

where $m_{{px}}$, $m_{{py}}$ and $m_{{pxy}}$ indicate the bending and torsional moment created by the piezoelectric actuators. Equation (3) can be rewritten as Eq. (4).

4 $$\left[ {D\nabla ^{4} w_{b} - J\left( {1 + \frac{{\bar{m}D}}{{JS}}} \right)\frac{{\partial ^{2} }}{{\partial t^{2} }}\left( {\frac{{\partial ^{2} w_{b} }}{{\partial x^{2} }} + \frac{{\partial ^{2} w_{b} }}{{\partial y^{2} }}} \right) + \bar{m}\frac{{\partial ^{2} w_{b} }}{{\partial t^{2} }} + \frac{{\bar{m}J}}{S}\frac{{\partial ^{4} w_{b} }}{{\partial t^{4} }}} \right] = \left[ {P\left( {x,y,t} \right) - \frac{{\partial ^{2} m_{{px}} }}{{\partial x^{2} }} - 2\frac{{\partial ^{2} m_{{pxy}} }}{{\partial x\partial y}} - \frac{{\partial ^{2} m_{{py}} }}{{\partial y^{2} }} + \frac{J}{{D\left( {1 + \upsilon } \right)}}\left( {1 + \frac{{\bar{m}D}}{{JS}}} \right)\frac{{\partial ^{2} }}{{\partial t^{2} }}\left( {m_{{px}} + m_{{py}} } \right)} \right].$$

The moments generated by the N piezoelectric actuators have the same thickness and properties subjected to a voltage can be calculated using Eq. (5)^22^.

5 $$\left\{ {\begin{array}{*{20}c} {m_{{px}} \left( {x,y,t} \right) = C_{0} \frac{{d_{{31}} }}{{h_{{PZT}} }}V\left( t \right)R_{p} ^{{n_{a} }} \left( {x,y} \right)} \\ {m_{{py}} \left( {x,y,t} \right) = C_{0} \frac{{d_{{32}} }}{{h_{{PZT}} }}V\left( t \right)R_{p} ^{{n_{a} }} \left( {x,y} \right)} \\ {m_{{pxy}} \left( {x,y,t} \right) = C_{0} \frac{{d_{{36}} }}{{h_{{PZT}} }}V\left( t \right)R_{p} ^{{n_{a} }} (x,y) = 0} \\ \end{array} } \right.$$

6 $$R_{p} ^{{n_{a} }} \left( {x,y} \right) = \left[ {H\left( {x - x_{1}^{{n_{a} }} } \right) - H\left( {x - x_{2}^{{n_{a} }} } \right)} \right]\left[ {H\left( {y - y_{1}^{{n_{a} }} } \right) - H\left( {y - y_{2}^{{n_{a} }} } \right)} \right]$$

where $d_{{31}} ,d_{{32}}$ and $d_{{36}} = 0$ are the strain constants of the piezoelectric materials, $h_{{PZT}}$ is patch thickness, $H\left( . \right)$ is Heaviside function, $x_{1}^{{n_{a} }}$ and $y_{1}^{{n_{a} }}$ indicate the coordinates of the bottom left corner of $n_{a}$th Piezoelectric patch, $x_{2}^{{n_{a} }}$ and $y_{2}^{{n_{a} }}$ indicate the coordinates of the top right corner of $n_{a}$th Piezoelectric patch, $V\left( t \right)$ is the voltage, and $C_{0}$ is obtained from the relation (7)^22^.

7 $$C_{0} = \frac{{ - 1}}{6}\frac{{1 + \upsilon _{{PZT}} }}{{1 - \upsilon }}\frac{{Eh^{2} K}}{{1 + \upsilon - \left( {1 + \upsilon _{{PZT}} } \right)K}}$$

where $\upsilon$ and $\upsilon _{{PZT}}$ are Poisson ratio of plate materials and piezoelectric materials, respectively. $K$ is given by^22^,

8 $$K = \frac{{ - E_{{PZT}} }}{E}\frac{{1 - \upsilon ^{2} }}{{1 - \upsilon _{{PZT}}^{2} }}\frac{{1.5h_{{PZT}} h\left( {h + h_{{PZT}} } \right)}}{{2\left( {0.125h^{3} + h_{{PZT}}^{3} } \right) + 1.5h \times h_{{PZT}}^{2} }}$$

where $E_{{PZT}}$ is the modulus of elasticity piezoelectric patches.

### Finite difference method

**Figure 2:**
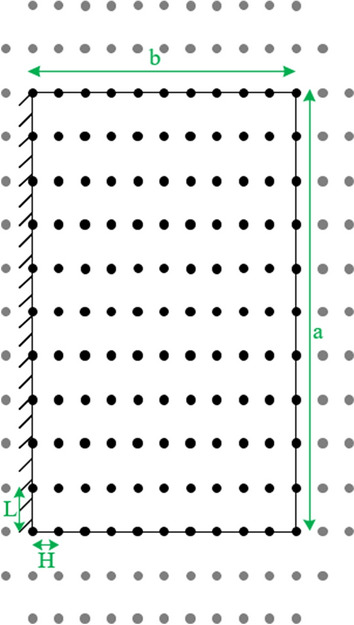
Original and additional nodes are represented by black and gray color, respectively.

**Figure 3:**
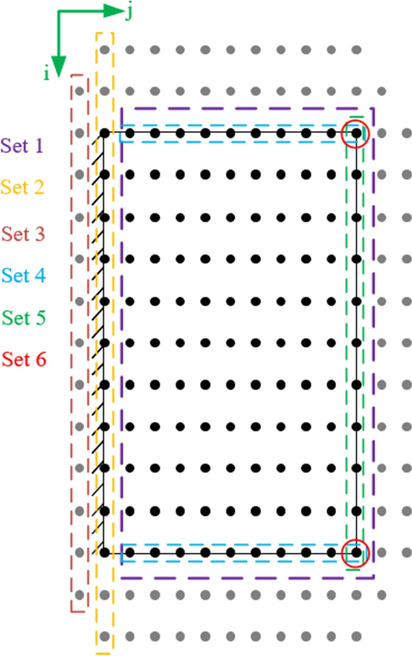
Node sets which include some nodes to assign by a number of equations in finite difference method.

The first stage in using the finite difference method is dividing the thick plate to a finite mesh of nodes with the horizontal and vertical distance between each node shown by H and L, respectively^22^. Also, additional knots outside the plate are used to show the boundary conditions on the cantilever thick plate in the finite difference method as shown in Fig. 2. In the next step, the nodes are divided to six sets (Fig. 3). Each set is determined by some equations explained in the following sections^22^.

#### Set 1 nodes

Each node in this set is represented by a relation obtained by Eq. (4), and terms related to the finite difference method in this equation are obtained from the following relationships.

9 $$\begin{aligned} \nabla ^{4} w_{b} \left( {i,j,t} \right) & \cong \left[ {\left[ {\frac{6}{{H^{4} }} + \frac{6}{{L^{4} }} + \frac{8}{{H^{2} L^{2} }}} \right]w_{b} \left( {i,j,t} \right) + \left[ {\frac{{ - 4}}{{H^{4} }} - \frac{{ - 4}}{{H^{2} L^{2} }}} \right]\left( {\begin{array}{*{20}c} {w_{b} \left( {i,j - 1,t} \right) + w_{b} \left( {i,j + 1,t} \right)} \\ { + w_{b} \left( {i - 1,j,t} \right) + w_{b} \left( {i + 1,j,t} \right)} \\ \end{array} } \right)} \right. \\ & \left. {\quad + \frac{2}{{H^{2} L^{2} }}\left( {\begin{array}{*{20}c} {w_{b} \left( {i - 1,j - 1,t} \right) + w_{b} \left( {i - 1,j + 1,t} \right)} \\ { + w_{b} \left( {i + 1,j - 1,t} \right) + w_{b} \left( {i + 1,j + 1,t} \right)} \\ \end{array} } \right) + \frac{1}{{H^{4} }}\left( {\begin{array}{*{20}c} {w_{b} \left( {i,j + 2,t} \right) + w_{b} \left( {i,j - 2,t} \right)} \\ { + w_{b} \left( {i - 2,j,t} \right) + w_{b} \left( {i + 2,j,t} \right)} \\ \end{array} } \right)} \right] \\ \end{aligned}$$

10 $$\frac{{\partial ^{2} w_{b} \left( {i,j,t} \right)}}{{\partial x^{2} }} = - \frac{1}{{H^{2} }}\left[ {w_{b} \left( {i,j - 1,t} \right) - 2w_{b} \left( {i,j,t} \right) + w_{b} \left( {i,j + 1,t} \right)} \right]$$

11 $$\frac{{\partial ^{2} w_{b} \left( {i,j,t} \right)}}{{\partial y^{2} }} = - \frac{1}{{L^{2} }}\left[ {w_{b} \left( {i - 1,j,t} \right) - 2w_{b} \left( {i,j,t} \right) + w_{b} \left( {i + 1,j,t} \right)} \right]$$

12 $$\frac{{\partial ^{2} m_{{px}} \left( {i,j,t} \right)}}{{\partial x^{2} }} \cong \frac{{ - C_{0} d_{{31}} }}{{h_{{PZT}} H^{2} }}V\left( t \right)\left[ {R^{{n_{a} }} \left( {i,j - 1} \right) - 2R^{{n_{a} }} \left( {i,j} \right) + R^{{n_{a} }} \left( {i,j + 1} \right)} \right]$$

13 $$\frac{{\partial ^{2} m_{{py}} \left( {i,j,t} \right)}}{{\partial y^{2} }} \cong \frac{{ - C_{0} d_{{32}} }}{{h_{{PZT}} L^{2} }}V\left( t \right)\left[ {R^{{n_{a} }} \left( {i - 1,j} \right) - 2R^{{n_{a} }} \left( {i,j} \right) + R^{{n_{a} }} \left( {i + 1,j} \right)} \right]$$

14 $$\frac{{\partial ^{2} m_{{pxy}} \left( {i,j,t} \right)}}{{\partial x\partial y}} \cong 0$$

i and j represent the row and column of the node, respectively, and t denotes time. By substituting relationships (9)–(14) in the relationship (4).

The time-varying dynamic load can be written as $P(x,y,t)=F(t)P(x,y)$ by the separation of variables.

15 $$\begin{aligned}  & \left[{\begin{array}{*{20}c} {\frac{{\bar{m}J}}{s}\ddddot w_{b} \left( {i,j,t} \right) + \bar{m}\ddot{w}_{b} \left( {i,j,t} \right) - J\left( {1 + \frac{{\bar{m}D}}{{JS}}} \right)\left( { - \frac{1}{{H^{2} }}\left( {\begin{array}{*{20}l} {\ddot{w}_{b} \left( {i,j - 1,t} \right) - 2\ddot{w}_{b} \left( {i,j,t} \right)} \hfill \\ {\quad + \ddot{w}_{b} \left( {i,j + 1,t} \right)} \hfill \\ \end{array} } \right) - \frac{1}{{L^{2} }}\left( {\begin{array}{*{20}l} {\ddot{w}_{b} \left( {i - 1,j,t} \right) - 2\ddot{w}_{b} \left( {i,j,t} \right)} \hfill \\ {\quad + \ddot{w}_{b} \left( {i + 1,j,t} \right)} \hfill \\ \end{array} } \right)} \right)}  \\&   + D\left( {\begin{array}{*{20}l} {\left[ {\frac{6}{{H^{4} }} + \frac{6}{{L^{4} }} + \frac{8}{{H^{2} L^{2} }}} \right]w_{b} \left( {i,j,t} \right) + \left[ {\frac{{ - 4}}{{H^{4} }} - \frac{{ - 4}}{{H^{2} L^{2} }}} \right]\left( {\begin{array}{*{20}c} {w_{b} \left( {i,j - 1,t} \right) + w_{b} \left( {i,j + 1,t} \right)} \\ { + w_{b} \left( {i - 1,j,t} \right) + w_{b} \left( {i + 1,j,t} \right)} \\ \end{array} } \right)} \hfill \\ {\quad + \frac{2}{{H^{2} L^{2} }}\left( {\begin{array}{*{20}c} {w_{b} \left( {i - 1,j - 1,t} \right) + w_{b} \left( {i - 1,j + 1,t} \right)} \\ { + w_{b} \left( {i + 1,j - 1,t} \right) + w_{b} \left( {i + 1,j + 1,t} \right)} \\ \end{array} } \right) + \frac{1}{{H^{4} }}\left( {\begin{array}{*{20}c} {w_{b} \left( {i,j + 2,t} \right) + w_{b} \left( {i,j - 2,t} \right)} \\ { + w_{b} \left( {i - 2,j,t} \right) + w_{b} \left( {i + 2,j,t} \right)} \\ \end{array} } \right)} \hfill \\ \end{array} } \right)\\ \end{array} } \right]\\& =F(t)P(i,j) - V(t)Z^{{n_{a} }} (i,j) \\ \end{aligned}$$

16 $$Z^{{n_{a} }} \left( {i,j} \right) = \frac{{ - C_{0} d_{{31}} }}{{h_{{PZT}} H^{2} }}\left[ {R_{p} ^{{n_{a} }} \left( {i,j - 1} \right) - 2R_{p} ^{{n_{a} }} \left( {i,j} \right) + R_{p} ^{{n_{a} }} \left( {i,j + 1} \right)} \right] + \frac{{ - C_{0} d_{{32}} }}{{h_{{PZT}} L^{2} }}\left[ {R_{p} ^{{n_{a} }} \left( {i - 1,j} \right) - 2R_{p} ^{{n_{a} }} \left( {i,j} \right) + R_{p} ^{{n_{a} }} \left( {i + 1,j} \right)} \right]$$

#### Set 2 nodes

The set of second nodes is related to the clamped edge of the thick plate. The relationship of each node in this set is calculated according to the displacement at the clamped edge^22^.

17 $$w_{b} \left( {i,j,t} \right) = 0$$

#### Set 3 nodes

The third nodes are the set of additional nodes on the left side of the clamped edge of the thick plate. To get the relationship of these nodes, the rotation around the clamped edge in boundary conditions is considered. Therefore, the relation of each node in this set is as follows^22^.

18 $$w_{b} \left( {i,j,t} \right) = w_{b} \left( {i,j + 2,t} \right)$$

#### Set 4 nodes

The fourth node set includes free nodes at the bottom and top edges of the plate. The boundary conditions at these edges are as follows^22^.

19 $$\left\{ {\begin{array}{*{20}c} {M_{y} \left( {x,y,t} \right) = - D\left[ {\frac{{\partial ^{2} w_{b} \left( {x,y,t} \right)}}{{\partial y^{2} }} + \nu \frac{{\partial ^{2} w_{b} \left( {x,y,t} \right)}}{{\partial x^{2} }}} \right] = 0{}} \\ {\bar{Q}_{y} \left( {x,y,t} \right) = Q_{y} + \frac{{\partial M_{{xy}} }}{{\partial x}} = - D\left[ {\begin{array}{*{20}c} {\frac{{\partial ^{3} w_{b} \left( {x,y,t} \right)}}{{\partial y^{3} }} + } \\ {\left( {2 - \nu } \right)\frac{{\partial ^{3} w_{b} \left( {x,y,t} \right)}}{{\partial x^{2} \partial y}}} \\ \end{array} } \right] = 0} \\ \end{array} } \right.$$

where $\bar{Q}_{y}$ is the shearing force which includes the effect of torsional moment and the transverse shear force. $Q_{y}$ is transverse shear force (see Supplementary Note).

Therefore, the following two equations must be satisfied for each node in this set.

20 $$\left[ {\left( { - 2 - 2\upsilon } \right)w_{b} \left( {i,j,t} \right) + \nu \left[ {w_{b} \left( {i,j - 1,t} \right) + w_{b} \left( {i,j + 1,t} \right)} \right] + w_{b} \left( {i - 1,j,t} \right) + w_{b} \left( {i + 1,j,t} \right)} \right] = 0$$

21 $$\left[ {\left( {2\upsilon - 6} \right)\left[ {w_{b} \left( {i - 1,j,t} \right) - w_{b} (i + 1,j,t} \right] + \left( {2 - \nu } \right)\left[ {\begin{array}{*{20}c} {w_{b} \left( {i - 1,j - 1,t} \right) + w_{b} \left( {i - 1,j + 1,t} \right)} \\ { - w_{b} \left( {i + 1,j - 1,t} \right) - w_{b} \left( {i + 1,j + 1,t} \right)} \\ \end{array} } \right] + w_{b} \left( {i - 2,j,t} \right) - w_{b} \left( {i + 2,j,t} \right)} \right] = 0$$

#### Set 5 nodes

The fifth node set includes the free edge nodes to the right of the plate. The boundary conditions at these edges are as follows^22^.

22 $$\left\{ {\begin{array}{*{20}c} {M_{x} \left( {x,y,t} \right) = - D\left[ {\frac{{\partial ^{2} w_{b} \left( {x,y,t} \right)}}{{\partial x^{2} }} + \nu \frac{{\partial ^{2} w_{b} \left( {x,y,t} \right)}}{{\partial y^{2} }}} \right] = 0{}} \\ {\bar{Q}_{x} \left( {x,y,t} \right) = Q_{x} + \frac{{\partial M_{{xy}} }}{{\partial y}} = - D\left[ {\begin{array}{*{20}c} {\frac{{\partial ^{3} w_{b} \left( {x,y,t} \right)}}{{\partial x^{3} }} + } \\ {\left( {2 - \nu } \right)\frac{{\partial ^{3} w_{b} \left( {x,y,t} \right)}}{{\partial x\partial y^{2} }}} \\ \end{array} } \right] = 0} \\ \end{array} } \right.$$

where ${\stackrel{-}{Q}}_{x}$ is the shearing force, including the effect of torsional moment and the transverse shear force. ${Q}_{x}$ is transverse shear force (see Supplementary Note).

Therefore, the following two equations must be satisfied for each node in this set.

23 $$\left[ {\left( { - 2 - 2\upsilon } \right)w_{b} \left( {i,j,t} \right) + \nu \left[ {w_{b} \left( {i - 1,j,t} \right) + w_{b} \left( {i + 1,j,t} \right)} \right] + w_{b} \left( {i,j - 1,t} \right) + w_{b} \left( {i,j + 1,t} \right)} \right] = 0.$$

24 $$\left[ {\left( {2\upsilon - 6} \right)\left[ {w_{b} \left( {i,j + 1,t} \right) - w_{b} (i,j - 1,t} \right] + \left( {2 -\nu} \right)\left[ {\begin{array}{*{20}c} {w_{b} \left( {i - 1,j + 1,t} \right) + w_{b} \left( {i + 1,j + 1,t} \right)} \\ { - w_{b} \left( {i - 1,j - 1,t} \right) - w_{b} \left( {i + 1,j - 1,t} \right)} \\ \end{array} } \right] + w_{b} \left( {i,j + 2,t} \right) - w_{b} \left( {i,j - 2,t} \right)} \right] = 0$$

#### Set 6 nodes

Finally, the sixth node set consists of two nodes in the corners of the free edge of the plate that must satisfy the following equation^22^.

25 $$R_{{cf}} = 2D\left( {1 - \nu } \right)\frac{{\partial ^{2} w_{b} \left( {x,y,t} \right)}}{{\partial x\partial y}} = 0$$

where $R_{{cf}}$ is the concentrated forces. The relationship of the finite difference is as follows.

26 $$\left[ {w_{b} \left( {i - 1,j + 1,t} \right) - w_{b} \left( {i - 1,j - 1,t} \right) + w_{b} \left( {i + 1,j - 1,t} \right) + w_{b} \left( {i + 1,j + 1,t} \right)} \right] = 0$$

### State-space model

Each node in the first set is denoted by the index from 1 to n, n is the summation of the nodes in the first set. By getting $R_{p} ^{{n_{a} }} \left( {x,y} \right)$ from the relation (6) and substituting it into relation (16) the value of $Z^{{n_{a} }}$ is obtained for each piezoelectric patch. The motion of each node in this set is calculated from Eq. (15), and it is related to mesh size, applied load, displacement of adjacent nodes, the properties of the thick plate, applied voltage by piezoelectric patches and $Z^{{n_{a} }}$ value in these nodes and adjacent node.

In addition, nodes in sets 2–6 are identified by indices from n + 1 to $n_{t}$. $n_{t}$ is the sum of all nodes. The displacement of each node in this set is obtained according to the relationships mentioned in the previous sections. This method leads to a linear system as follows.

Where $0$ and $I$ show the zero and identity matrices, respectively. $n_{{ad}} = n_{t} - n$ is the number of additional nodes (ad)^22^. The variables $AM$ can be calculated from Eq. (15) for each node. The variables $AC11,{\text{~}}AC12,{\text{~}}AC21,$ and $AC22$ are coefficients of $w$ in the sets 1–6.

The condensed matrix can be calculated by removing the extra nodes. The reduced system is as follows^20^.

27 $$\begin{aligned} & \left[ {\begin{array}{*{20}c} {\left( {\frac{{\bar{m}J}}{s}} \right)I_{{(n \times n)}} } & {0_{{(n \times n_{{ad}} )}} } \\ {0_{{(n_{{ad}} \times n)}} } & {0_{{(n_{{ad}} \times n_{{ad}} )}} } \\ \end{array} } \right]\left[ {\begin{array}{*{20}c} {\begin{array}{*{20}c} {\begin{array}{*{20}c} {\begin{array}{*{20}c} {\ddddot w_{1} } \\ {\ddddot w_{2} } \\ \end{array} } \\ \vdots \\ \end{array} } \\ {\ddddot w_{n} } \\ \end{array} } \\ {\ddddot w_{{n + 1}} } \\ \vdots \\ {\ddddot w_{{n_{t} }} } \\ \end{array} } \right] + \left[ {\begin{array}{*{20}c} {AM_{{\left( {n \times n} \right)}} } & {0_{{\left( {n \times n_{{ad}} } \right)}} } \\ {0_{{\left( {n_{{ad}} \times n} \right)}} } & {0_{{\left( {n_{{ad}} \times n_{{ad}} } \right)}} } \\ \end{array} } \right]\left[ {\begin{array}{*{20}c} {\begin{array}{*{20}c} {\begin{array}{*{20}c} {\begin{array}{*{20}c} {\ddot{w}_{1} } \\ {\ddot{w}_{2} } \\ \end{array} } \\ \vdots \\ \end{array} } \\ {\ddot{w}_{n} } \\ \end{array} } \\ {\ddot{w}_{{n + 1}} } \\ \vdots \\ {\ddot{w}_{{n_{t} }} } \\ \end{array} } \right] \\ & \quad + \left[ {\begin{array}{*{20}c} {AC11_{{\left( {n \times n} \right)}} } & {AC12_{{\left( {n \times n_{{ad}} } \right)}} } \\ {AC21_{{\left( {n_{{ad}} \times n} \right)}} } & {AC22_{{\left( {n_{{ad}} \times n_{{ad}} } \right)}} } \\ \end{array} } \right]\left[ {\begin{array}{*{20}c} {\begin{array}{*{20}c} {\begin{array}{*{20}c} {\begin{array}{*{20}c} {w_{1} } \\ {w_{2} } \\ \end{array} } \\ \vdots \\ \end{array} } \\ {w_{n} } \\ \end{array} } \\ {w_{{n + 1}} } \\ \vdots \\ {w_{{n_{t} }} } \\ \end{array} } \right] = F(t)\left[ {\begin{array}{*{20}c} {\begin{array}{*{20}c} {\begin{array}{*{20}c} {\begin{array}{*{20}c} {P_{1} } \\ {P_{2} } \\ \end{array} } \\ \vdots \\ \end{array} } \\ {P_{n} } \\ \end{array} } \\ 0 \\ \vdots \\ 0 \\ \end{array} } \right] - V(t)\left[ {\begin{array}{*{20}c} {Z_{1}^{1} } & \cdots & {Z_{1}^{{n_{a} }} } \\ {Z_{2}^{1} } & \cdots & {Z_{2}^{{n_{a} }} } \\ \vdots & \cdots & \vdots \\ {Z_{n}^{1} } & \cdots & {Z_{n}^{{n_{a} }} } \\ 0 & \cdots & 0 \\ \vdots & \cdots & \vdots \\ 0 & \cdots & 0 \\ \end{array} } \right] \\ \end{aligned}$$

28 $$T = \left[ {\begin{array}{*{20}c} {I_{{\left( {n \times n} \right)}} } \\ { - AC22^{{ - 1}} \times AC21} \\ \end{array} } \right]$$

29 $$AC_{{n \times n}} = T^{T} \times \left[ {\begin{array}{*{20}c} {AC11_{{\left( {n \times n} \right)}} } & {AC12_{{\left( {n \times n_{{ad}} } \right)}} } \\ {AC21_{{\left( {n_{{ad}} \times n} \right)}} } & {AC22_{{\left( {n_{{ad}} \times n_{{ad}} } \right)}} } \\ \end{array} } \right] \times T$$

30 $$\left( {\frac{{\bar{m}J}}{s}} \right)_{{\left( {n \times n} \right)}} \left[ {\begin{array}{*{20}c} {\ddddot w_{1} } \\ {\ddddot w_{2} } \\ \vdots \\ {\ddddot w_{n} } \\ \end{array} } \right] + AM_{{\left( {n \times n} \right)}} \left[ {\begin{array}{*{20}c} {\ddot{w}_{1} } \\ {\ddot{w}_{2} } \\ \vdots \\ {\ddot{w}_{n} } \\ \end{array} } \right] + AC_{{\left( {n \times n} \right)}} \left[ {\begin{array}{*{20}c} {w_{1} } \\ {w_{2} } \\ \vdots \\ {w_{n} } \\ \end{array} } \right] = F\left( t \right)\left[ {\begin{array}{*{20}c} {P_{1} } \\ {P_{2} } \\ \vdots \\ {P_{n} } \\ \end{array} } \right] - V\left( t \right)\left[ {\begin{array}{*{20}c} {Z_{1}^{1} } & \cdots & {Z_{1}^{{n_{a} }} } \\ {Z_{2}^{1} } & \cdots & {Z_{2}^{{n_{a} }} } \\ \vdots & \cdots & \vdots \\ {Z_{n}^{1} } & \cdots & {Z_{n}^{{n_{a} }} } \\ \end{array} } \right]$$

Assuming state variables as $x\left( t \right) = \left[ {w_{1} ,...,w_{n} ,\dot{w}_{1} ,...,\dot{w}_{n} ,\ddot{w}_{1} ,...,\ddot{w}_{n} ,\dddot w_{1} ,...,\dddot w_{n} } \right]^{T}$, the state space form is calculated as follows.

31 $$\begin{array}{*{20}c} {\dot{x}\left( t \right) = A_{s} x\left( t \right) + B_{s} \left\{ {F\left( t \right)P - V\left( t \right)Z} \right\}} \\ {y\left( t \right) = C_{s} x\left( t \right) + D_{s} \left\{ {F\left( t \right)P - V\left( t \right)Z} \right\}} \\ \end{array}$$

where

32 $$A_{s} = \left[ {\begin{array}{*{20}c} {0_{n} } & {I_{n} } & {0_{n} } & {0_{n} } \\ {0_{n} } & {0_{n} } & {I_{n} } & {0_{n} } \\ {0_{n} } & {0_{n} } & {0_{n} } & {I_{n} } \\ { - \left( {\frac{s}{{\bar{m}J}}} \right)AC} & {0_{n} } & { - \left( {\frac{s}{{\bar{m}J}}} \right)AM} & {0_{n} } \\ \end{array} } \right],\;B_{s} = \left[ {\begin{array}{*{20}c} {\begin{array}{*{20}c} {0_{n} } \\ {0_{n} } \\ \end{array} } \\ {0_{n} } \\ {\left( {\frac{s}{{\bar{m}J}}} \right)} \\ \end{array} } \right],\;C_{s} = \left[ {\begin{array}{*{20}c} {0_{n} } & {I_{n} } & {\begin{array}{*{20}c} {0_{n} } & {0_{n} } \\ \end{array} } \\ \end{array} } \right],\;D_{s} = \left[ {0_{n} } \right]$$

The control force from actuators is calculated by the control matrix $B_{c} = B_{s} Z$, and $u = V\left( t \right)$ is control voltage of actuators $$V\left( t \right) = \left[ {\begin{array}{*{20}c} {V_{1} } & {V_{2} } & \cdots & {V_{{n_{a} }} } \\ \end{array} } \right]^{T}$$.

On the other hand, the piezoelectric sensor voltage is equal to^23^.

33 $$V_{s} \left( t \right) = {\text{R}}_{p} r^{{n_{a} }} \mathop \smallint \limits_{{y_{1}^{{n_{a} }} }}^{{y_{2}^{{n_{a} }} }} \mathop \smallint \limits_{{x_{1}^{{n_{a} }} }}^{{x_{2}^{{n_{a} }} }} \left( {e_{{31}} \frac{{\partial ^{2} \dot{w}}}{{\partial x^{2} }} + e_{{32}} \frac{{\partial ^{2} \dot{w}}}{{\partial y^{2} }} + 2e_{{36}} \frac{{\partial ^{2} \dot{w}}}{{\partial x\partial y}}} \right)dxdy$$

where ${\text{R}}_{p}$ and $r^{{n_{a} }}$ are the constant of the current amplifier and the distance between the middle plans of the plate and the sensor, respectively. $e_{{31}} ,{}e_{{32}}$ and $e_{{36}} = 0$ are the piezoelectric material stress constants of the piezoelectric patches^23^. By considering the same mesh as mentioned before and applying finite difference method, Eq. (33) can be rewritten as

34 $$V_{s} ^{{n_{a} }} \left( t \right) \cong {\text{R}}_{p} r^{{n_{a} }} \mathop \smallint \limits_{{y_{1}^{{n_{a} }} }}^{{y_{2}^{{n_{a} }} }} \mathop \smallint \limits_{{x_{1}^{{n_{a} }} }}^{{x_{2}^{{n_{a} }} }} \left\{ {\frac{{e_{{31}} }}{{H^{2} }}\left[ {\dot{w}\left( {i,j - 1,t} \right) - 2\dot{w}\left( {i,j,t} \right) + \dot{w}\left( {i,j + 1,t} \right)} \right] + e_{{32}} \left[ {\dot{w}\left( {i - 1,j,t} \right) - 2\dot{w}\left( {i,j,t} \right) + \dot{w}\left( {i + 1,j,t} \right)} \right]} \right\}dxdy$$

Devoting the nodes from 1 to n can be simplified the Eq. (34).

35 $$V_{s} ^{{n_{a} }} \left( t \right) \cong {\text{R}}_{p} r^{{n_{a} }} N_{{\left( {n_{a} \times n} \right)}} S_{{\left( {n \times n} \right)}} \left[ {\begin{array}{*{20}c} {\dot{w}_{1} } & {\dot{w}_{2} } & \cdots & {\dot{w}_{n} } \\ \end{array} } \right]^{T}$$

where $S$ is the coefficients calculated by finite difference method. The constant matrix $N$ indicates the weights related to the numerical integration for the elements which are covered by piezoelectric sensors, and the other elements are zeros.

The modified state-space output $y_{V}$ can be rewritten as

36 $$y_{V} \left( t \right) = C_{c} x\left( t \right) + D_{s} \left\{ {F\left( t \right)P - V\left( t \right)Z} \right\}$$

where $$C_{c} = {\text{R}}_{p} r^{{n_{a} }} N_{{(n_{a} \times n)}} S_{{(n \times n)}} C_{s}$$.

The active control gain can be determined by using LQR optimal control theory. Minimization of the performance index $J$ used for finding the optimal placements and optimal numbers.

37 $$J = \frac{1}{2}\mathop \smallint \limits_{0}^{\infty } \left( {\left\{ x \right\}^{T} \left[ Q \right]\left\{ x \right\} + \left\{ u \right\}^{T} \left[ R \right]\left\{ u \right\}} \right)dt$$

The dimensions of the matrices $\left[ Q \right]$ and $\left[ R \right]$ are equal to the number of state variables and piezoelectric actuators, respectively.

The control law is given by assuming full state feedback.

38 $$\left\{ u \right\} = - \left[ K \right]\left\{ x \right\}$$

with constant control gain

39 $$\left[ K \right] = \left[ R \right]^{{ - 1}} \left[ {B_{c} } \right]^{T} \left[ S \right]$$

By solving the Riccati equation given by below equation, $\left[ S \right]$ can be calculated.

40 $$\left[ {A_{s} } \right]^{T} \left[ S \right] + \left[ S \right]\left[ {A_{s} } \right] + \left[ Q \right] - \left[ S \right]\left[ {B_{c} } \right]\left[ R \right]^{{ - 1}} \left[ {B_{c} } \right]^{T} \left[ S \right] = 0$$

The system will be stable when $\left[ S \right]$ is positive definite.

### Singular value decomposition (SVD)

The SVD can factorize any matrix $B_{c}$ into three matrices. A diagonal matrix $\sigma$ with the same dimension as $B_{c}$ whose diagonal elements are the singular value of $B_{c}$ in a descending order. $M$ and $N$ are both unitary matrices ($M^{T} M = I$ and $NN^{T} = I$)^24^.

41 $$B_{c} = M\sigma N^{T}$$

Wang and Wang^24^ presented a controllability index for finding the optimal location of actuators by maximizing the global control force. Singular values can be considered as controllability index which shows the amount of control energy provided by the piezoelectric actuators on the plate for a certain control input. $\sigma _{k}$ is referred to as the kth degree of controllability of the system. The size and location of the piezoelectric actuators can be found by the magnitude of $\sigma _{k}$. A controllability index $\Omega$ is^24^:

42 $$\Omega = max_{{t = 1,N_{{ap}} }} \left( {\mathop \prod \limits_{k}^{{n_{a} }} \sigma _{k} } \right)$$

where $N_{{ap}}$ is the number of the existing positions and $n_{a}$ are the number of the actuators.

#### Norm of SVD

To find the optimal locations of the actuators, the norm of SVD has been maximized^1^. The objective function is indicated as

43 $$\Omega = max_{{t = 1,N_{{ap}} }} \left( {max_{{k = 1,n_{a} }} \sigma _{k} } \right)$$

#### The modified control matrix and singular value decomposition (MCSVD)

The rows and columns of the control matrix $B_{c}$ demonstrate the states of the system and the number of actuators as inputs that are given to the system, respectively. In the MCSVD approach, the singular values of the column control matrix are taken as the fitness function because of increasing closed-loop average dB gain, and also optimal positions of the sensor and actuators are found by maximizing the fitness function^1,25^. The number of actuators on the plate are composed to create a large size actuator by collecting the columns of the control matrix to form a column matrix^1,25^. The singular value of the column matrix is dependent on locations which is provided by a combined patch (Eq. (44)) to form of a column matrix (Eq. (45)).

44 $$B_{c} = \left[ {\begin{array}{*{20}c} 0 & \cdots & 0 \\ \vdots & \cdots & \vdots \\ 0 & \cdots & 0 \\ {B_{{11}} } & \cdots & {B_{{1n_{a} }} } \\ \vdots & \cdots & \vdots \\ {B_{{n1}} } & \cdots & {B_{{nn_{a} }} } \\ \end{array} } \right]_{{4n \times n_{a} }}$$

45 $$B_{{c1}} = \left[ {\begin{array}{*{20}c} 0 \\ \vdots \\ 0 \\ {B_{{11}} + B_{{12}} + \cdots + B_{{1n_{a} }} } \\ \vdots \\ {B_{{n1}} + B_{{n2}} + \cdots + B_{{nn_{a} }} } \\ \end{array} } \right]_{{4n \times 1}}$$

The singular value of the matrix $B_{{c1}}$ is equal to the square roots of the eigenvalues of the matrix $B_{{c1}} B_{{c1}}^{T}$.

46 $$\Omega = max_{{t = 1,N_{{ap}} }} \left( {\sqrt {\left( {eig\left( {\left[ {B_{{c1}} } \right]{\text{*}}\left[ {B_{{c1}} } \right]^{T} } \right)} \right)} } \right)$$

The optimal location of piezoelectric patches are obtained by maximizing the Eq. (46) known as the controllability index that was calculated by maximizing the global force with binary-coded Genetic algorithm.

### 1-bit coding plate by genetic algorithm

**Figure 4:**
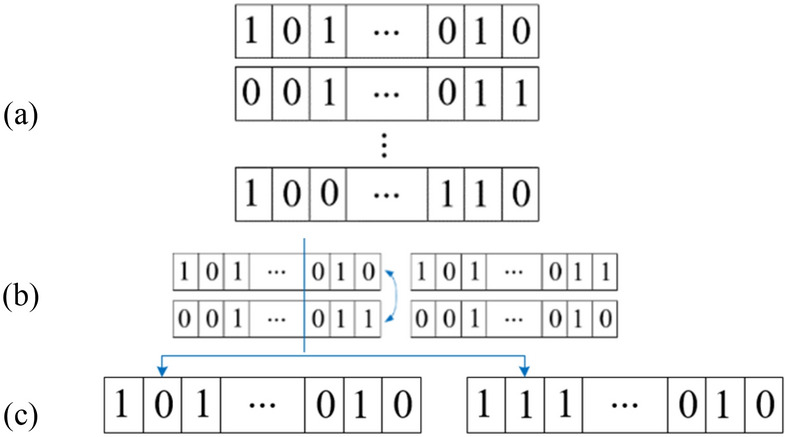
(**a**) Binary chromosome, (**b**) crossover, (**c**) mutation.

1-bit Coding plate is composed of only two kinds of unit cells with ‘0’ and ‘1’ response. When an actuator is not installed on a position, the coding state is ‘0’ and otherwise, it is ‘1’. Therefore, the chromosome is characterized by a binary demonstration of a combination of actuators. Figure 4 illustrates the binary chromosome, the crossover, and the punctual mutation. The binary code length is based on the number of elements of structures on the plate, which has been considered to be 100.

Many strategies have been used to apply the reproduction process, but all involve “crossover” and “mutation”. In a crossover, new chromosomes are produced by swapping pieces of parent’s chromosomes. One or more digits in a chromosome are changed randomly in mutation^1^. In this way, crossover is used to explore the known areas of the search space by trial different combinations of digits, while mutation maintains variety in the population and so to explore new areas of the search space and prevent the process from getting trapped in a local optimal solution^4^. Moreover, ensuring that two individuals generated are not allowed having the same values in given domain for the present case with crossover and mutation. Therefore, a constraint has been set to get unique individuals.

It is required to verify whether the individuals generated are valid after the genetic operations. In other words, it is significant to check whether all the binary representations gained after the crossover and the mutation follow the number of active elements which are between one and a maximum number desired. The binary representation sometimes is invalid because the number of actuators was not according to this maximum number (10 in this problem). So, this binary number must be replaced. If invalid individuals appear, it is essential to replace them to valid individuals. In the case of invalid individuals, these are changed by valid binary representations randomly selected. First, the values of each gene on each chromosome are summed with each other. If the sum is different from the total number of actuators mentioned, a valid representation is selected randomly. Thus, valid genes will always be obtained.

The GA process in this paper is given as follows:

1. The initial population is created randomly which the length of chromosome is equal to the total number of elements on the pate (i.e. 100).
2. A fitness value is computed for population members based on using a maximization problem instead of the minimization problem with the fitness −Ω according to Eq. (46). With a constraint which two gens of generated population are not allowed having same value and gens have absolute value.
3. The chromosomes are sorted according to their fitness value (controllability index).
4. Parents are selected, based on the highest fitness value.
5. Some individuals in the current population which have higher value of fitness are selected as elite to transfer to the next population.
6. Generate a new offspring by using the crossover on the selected parents (the crossover point being selected randomly).
7. Mutate one or two digits of the child’s chromosome with certain probability.
8. Evaluate the fitness value for new population members, and find the best one.
9. Feasibility test: go to step 11 if there are infeasible individuals. Otherwise, go to step 10.
10. Change the non-feasible individuals into feasible individuals and go to step 11.
11. If the predefined stop-condition is satisfied, the GA iteration is stopped, and returns the best solution in the current population.
12. Otherwise, go back to step 2. Put the previous result in the initial random population and repeat the process to obtain the global optimum.

**Figure 5:**
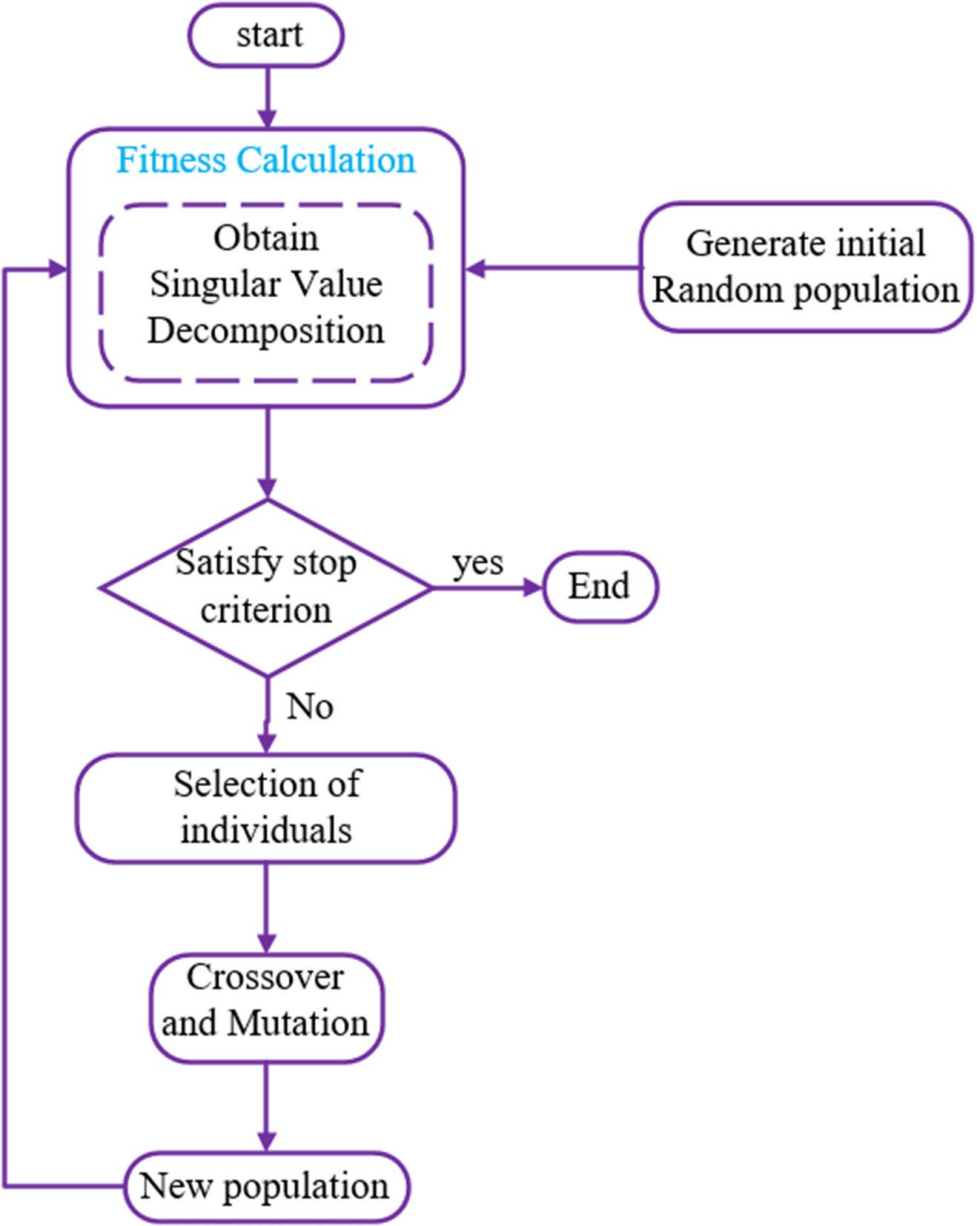
The flowchart of the genetic algorithm (GA).

The coding plate was founded by the MCSVD approach to suppress the first six modes. Figure 5 presents the flowchart of a GA for finding optimal number and placement of piezoelectric patches on the cantilever thick plate for suppressing the first six modes.

For the problem at hand, the ending condition of the GA is chosen to reach a certain number of iterations. For optimization, the configuration of GA in this work is considered as: crossover rate: 0.8, Stopping criterion: when the largest cost of the chromosomes cannot be further increased in 50 generations.

### Average closed-loop dB gain reduction

**Figure 6:**
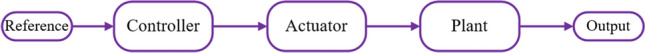
General open-loop control system.

**Figure 7:**
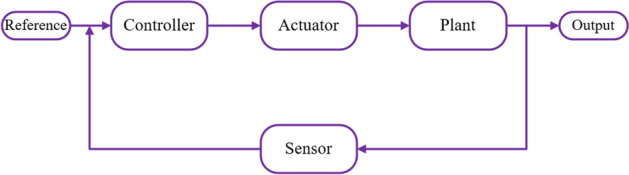
General closed-loop control system.

This method is applied to validate the optimal placements of piezoelectric patches obtained. Figures 6 and 7 show the open-loop (The control is performed without monitoring its output as there is no feedback) and closed-loop (The control is performed with sensors, composing a feedback that closes the loop) control.

The open-loop gain in dB is^26^:

47 $$GO_{{k,t_{m} }} = 20\log _{{10}} \left( {\left| {C_{{c}_{k}} \left( {j\omega _{{t_{m} }} I - A_{s} } \right)^{{ - 1}} B_{{c} _{k}} } \right|} \right)$$

where $t_{m}$ represents the specified natural mode at the sensor number $k~$ when a unite voltage used to the first actuator. $A_{s}$, $B_{{c} _{k}}$ and $C_{{c}_{k}}$ calculate by Eqs. (32) and (36).

By apply the sinusoidal unit voltage at a specified single actuator for all of the sensors and modes, the average open-loop dB gain is^26^:

48 $$MGO = \frac{1}{{n_{m} n_{a} }}\mathop \sum \limits_{{t_{m} = 1}}^{{n_{m} }} \mathop \sum \limits_{{k = 1}}^{{n_{a} }} GO_{{k,t_{m} }}$$

The closed-loop state matrix is:

49 $$A_{{C} _{s}} = A_{s} - B_{{c} _{k}} K_{k}$$

The closed-loop dB gain $i~$ is^26^:

50 $$GC_{{k,t_{m} }} = 20\log _{{10}} \left( {\left| {C_{{c}_{k}} \left( {j\omega _{{t_{m} }} I - A_{{C} _{s}} } \right)^{{ - 1}} B_{{c} _{k}} } \right|} \right)$$

The mean average closed-loop dB gain for different values of linear quadratic matrices is^26^:

51 $$MGC = \left. {\frac{1}{{3n_{m} n_{a} }}\left| {\mathop \sum \limits_{{q = 1}}^{3} \mathop \sum \limits_{{t_{m} = 1}}^{{n_{m} }} \mathop \sum \limits_{{k = 1}}^{{n_{a} }} GC_{{k,t_{m} }} } \right|} \right|_{{\begin{array}{*{20}c} {R = 1} \\ {Q_{k} = 10^{6} ,10^{7} ,10^{8} } \\ \end{array} }}$$

The average closed-loop dB gain reduction is defined as:

52 $$MGR = \left| {MGC - MGO} \right|$$

## Results and discussion

The properties and dimensions of the cantilever thick plate and piezoelectric patches are given in Table 1.

**Table 1:** Geometrical and physical data of the plate with the piezoelectric patches^27^.

Parameter	PZT-5H	Plate
x-Length [m]	0.1	1
y-Length [m]	0.05	0.5
Thickness [m]	0.005	0.06
${{\uprho}}\,{\text{[kg/m}}^{3} ]$	–	2770
${\text{E}}\,{\text{[Gpa]}}$	–	70
${{\upnu}}\,{\text{[}} - ]$	–	0.03
${{\upalpha }}_{{\text{M}}} = {{\upbeta }}_{{\text{K}}}$	–	5 × 10^−5^
${\text{C}}_{{11}} = {\text{C}}_{{22}} = {\text{C}}_{{33}} ,{\text{C}}_{{12{}}} \;[{\text{GPa}}]$	126.0, 79.5	–
${\text{C}}_{{13}} = {\text{C}}_{{23}} ,{\text{C}}_{{44}} = {\text{C}}_{{55}} = {\text{C}}_{{66{}}} \;[{\text{GPa}}]$	84.1, 23.0	–
${{\upchi}}_{{11}} = {{\upchi}}_{{22}} ,~{{\upchi }}_{{33}} \;[{\text{F/m}}]$	1.503 × 10^−8^, 1.30 × 10^−8^	–
${\text{e}}_{{15}} = {\text{e}}_{{24}} ,{\text{e}}_{{31}} = {\text{e}}_{{32}} ,{\text{e}}_{{33{}}} \;[{\text{C/m}}^{2} ]$	17, − 6.5, 23.3	–

**Figure 8:**
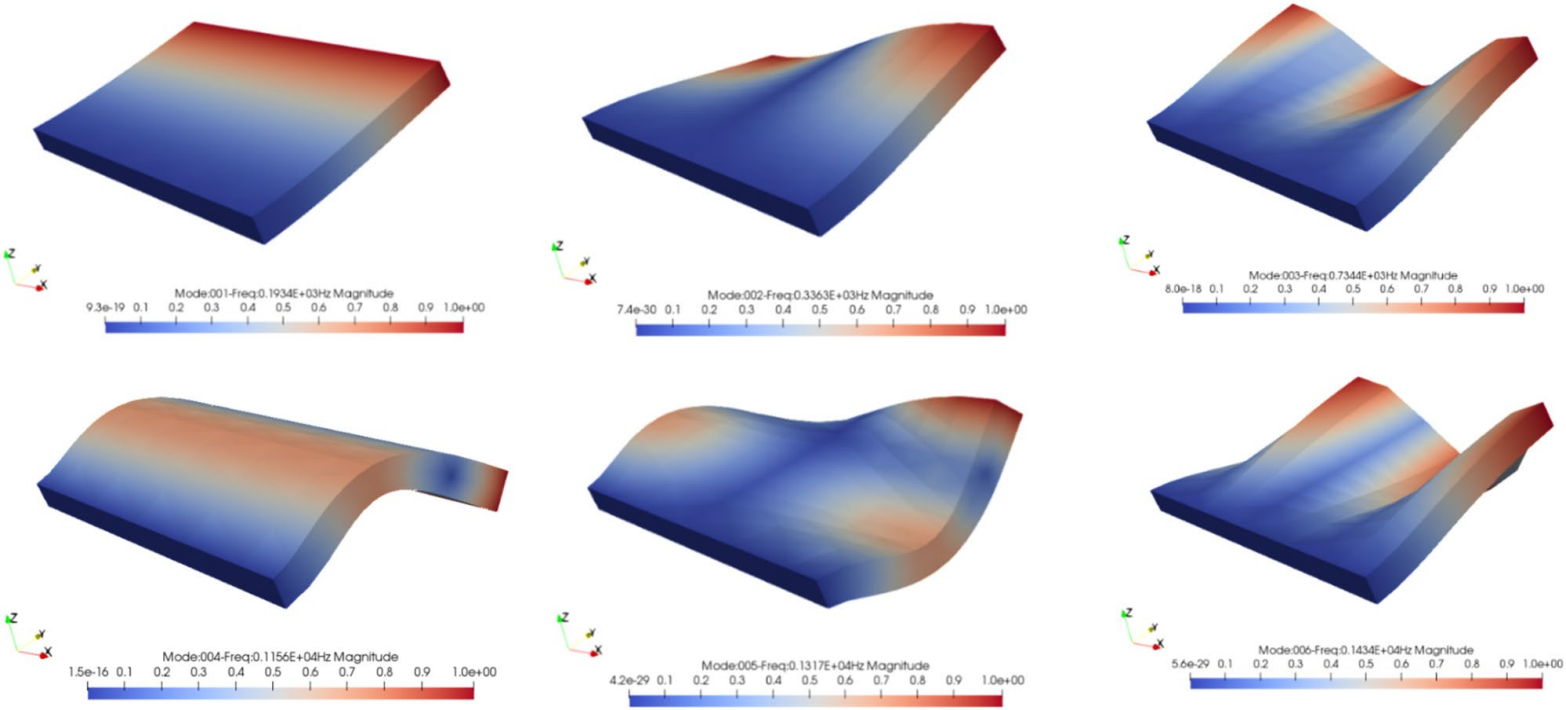
The first six modal shapes of a thick cantilever plate by FORTRAN.

The first resonance modes of the structure were obtained by applying the eigenvalue algorithm to the FDM model. The frequencies and shapes associated with some of these are presented in Table 2 and Fig. 8. It should be noted that the reason for the difference between the frequencies obtained between MATLAB and Abacus software in Table 2 is the difference between the number of elements used for modeling. Figure 8 illustrates that the shapes of the first six modes are symmetrically distributed about the plane of symmetry of the plate, which is perpendicular to its fixed end.

**Table 2:** The first six natural frequencies of the FDM model obtained through MATLAB codes, and ABAQUS.

Natural frequencies (Hz)	MATLAB	ABAQUS
Frequency 1	193.1245	189.18
Frequency 2	315.1004	308.61
Frequency 3	598.7914	576.30
Frequency 4	1147.72	1040.3
Frequency 5	1169.909	1125.2
Frequency 6	1304.712	1277.3

**Figure 9:**
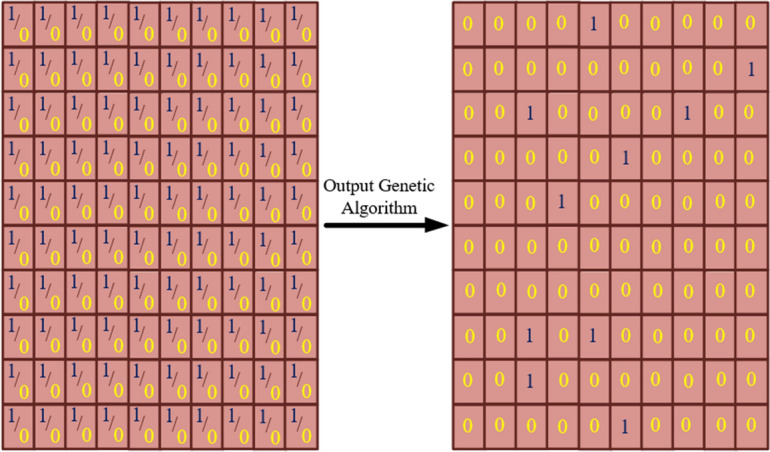
The piezoelectric output array of genetic algorithm.

**Figure 10:**
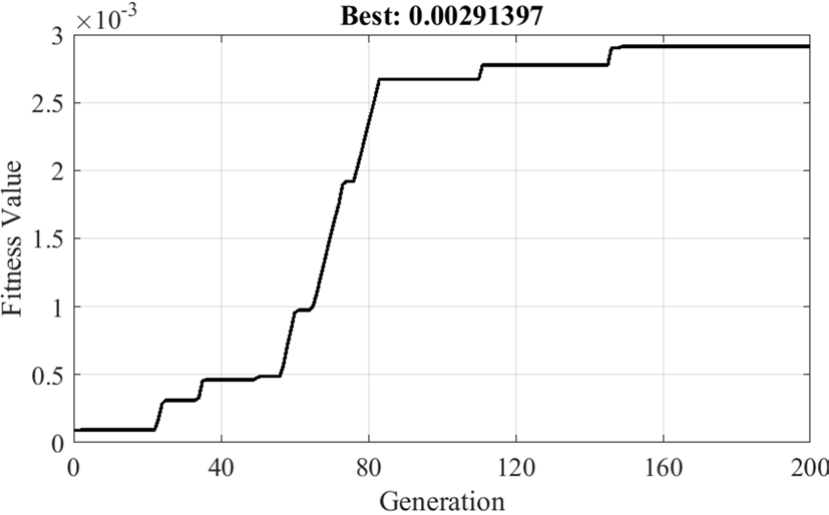
The convergence of the fitness value.

The cantilever rectangular thick plate was discretized in 10 × 10 quadrangular elements after optimization by GA which shows ten patches from the list of binary representations (possible solutions between one to ten patches on the plate) after terminating the conditions (Fig. 9). Moreover, the goal is not to find symmetrical places. The convergence of the fitness function for the case of cantilever thick plate indicates in Fig. 10.

**Figure 11:**
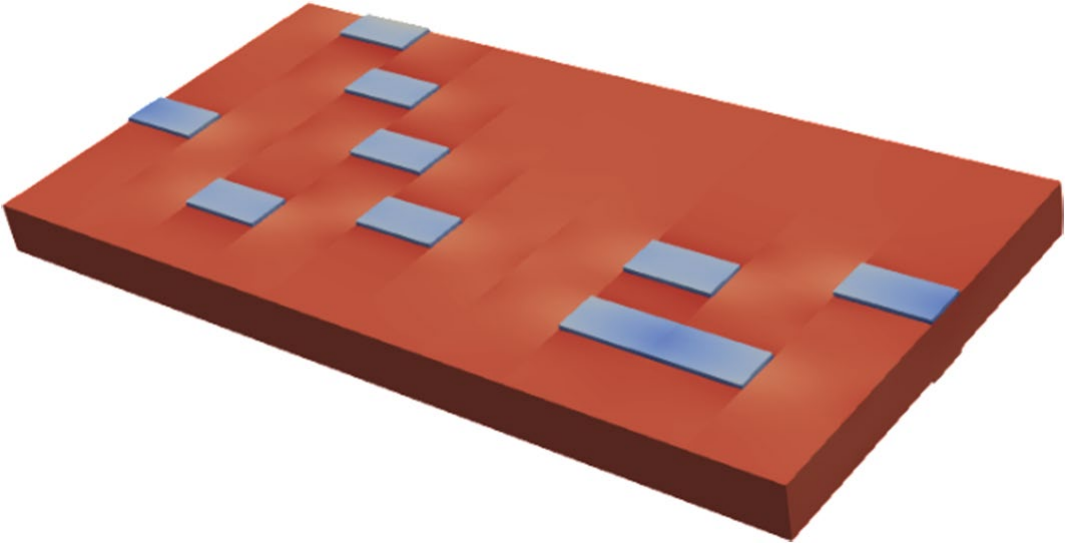
Ten piezoelectric patches on a cantilever thick plate by FORTRAN.

The ParaView representation of the thick plate with piezoelectric patches can be found in Fig. 11. Discrete piezoelectric pairs (actuator/sensor) give high actuating and sensing effects if they are appropriately located.

**Figure12:**
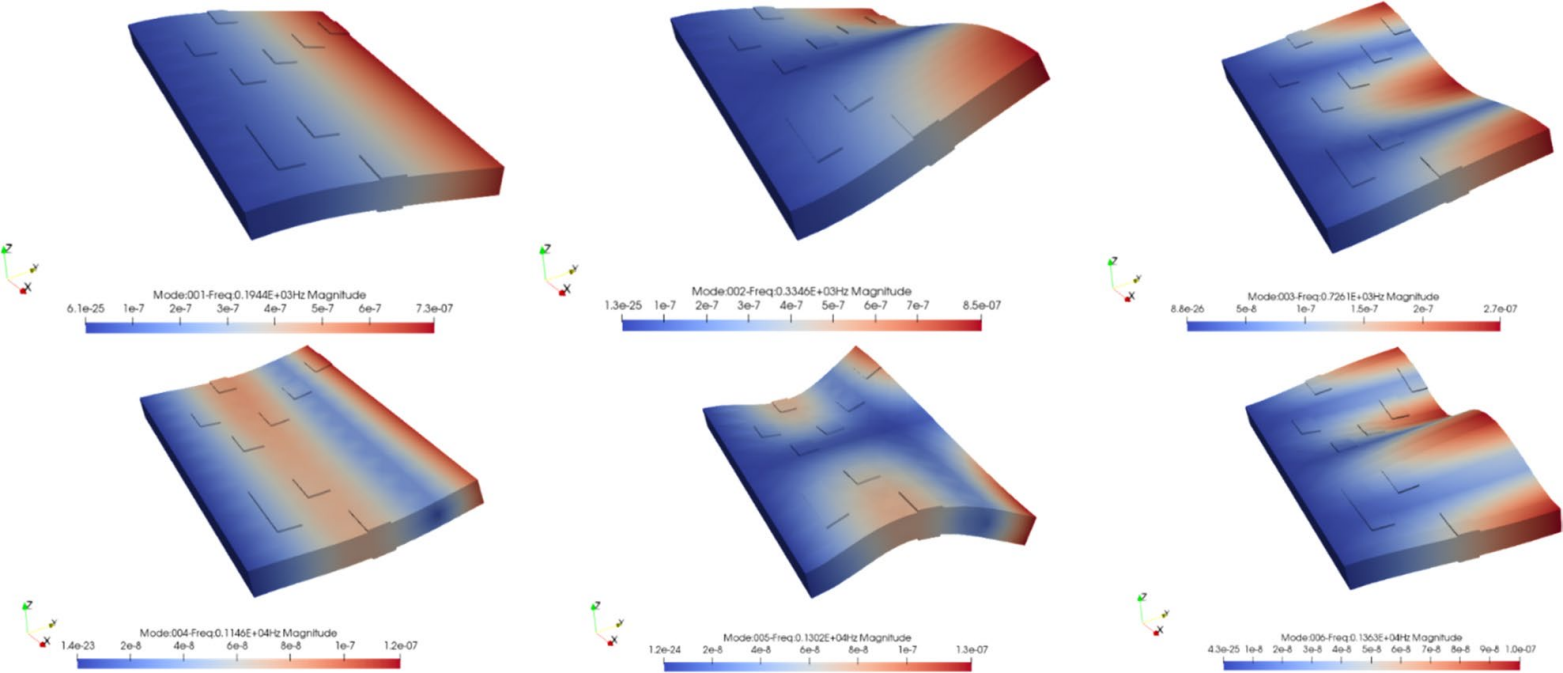
The first six modal shapes of the plate by FORTRAN.

Figure 12 shows the first six mode shapes of the thick plate with ten piezoelectric pairs, which are symmetrically distributed like Fig. 8.

**Figure 13:**
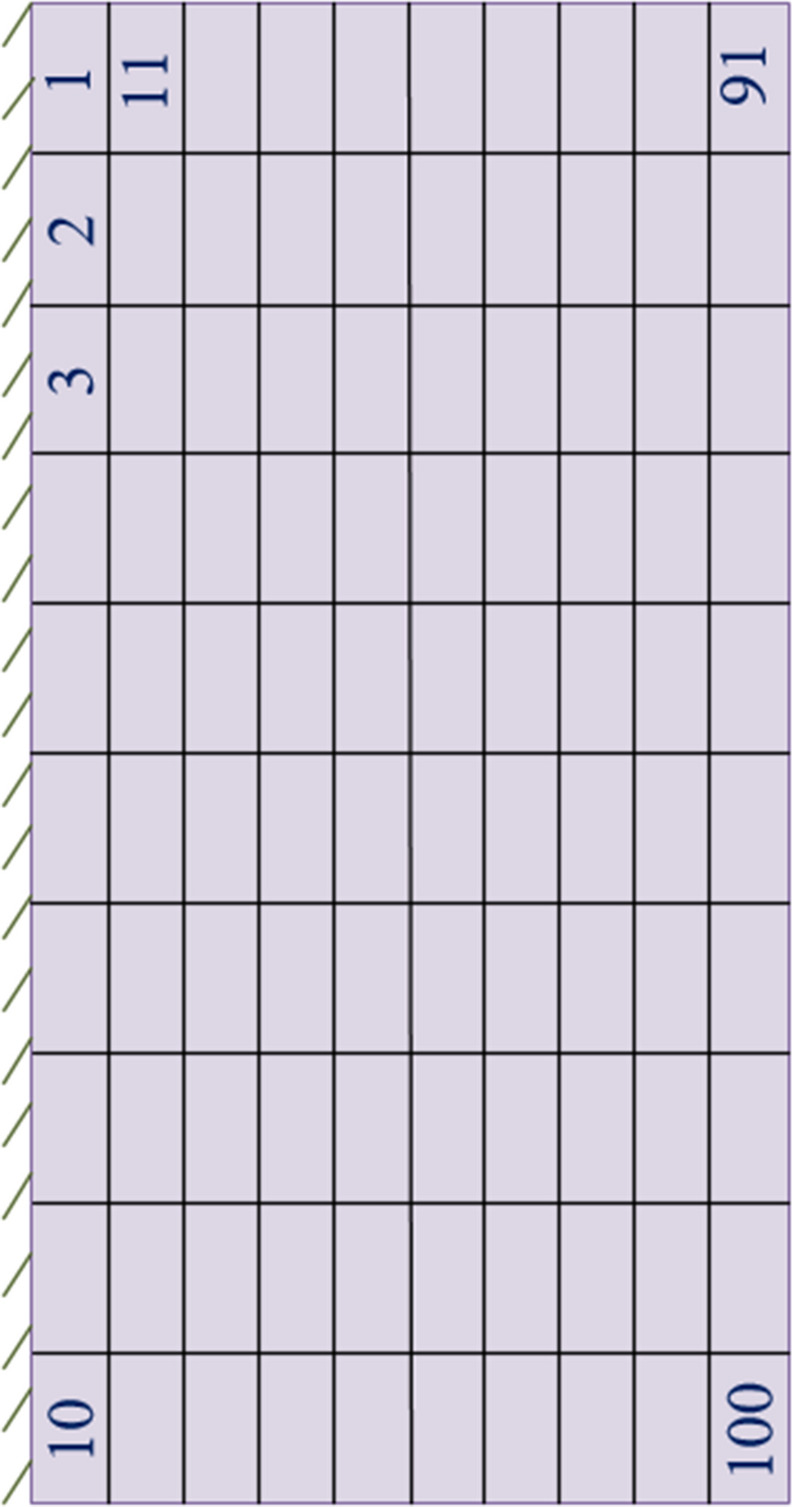
Cantilever smart plate having 100 elements.

The location of piezoelectric patches for each case are given in Table 3 based on the element number on the thick plate, which are shown in Fig. 13.

**Table 3:** Location of piezoelectric patches in different cases.

Ten S/A pairs	Element number based on Fig. 13									
Present study (Eq. (46))	23	28	29	35	41	48	54	60	73	92
Present study (Eq. (43))	28	33	35	37	43	46	48	54	75	77
Ref.^18^	1	2	5	9	11	36	45	56	95	96
Random	23	28	57	63	64	67	68	75	76	90

The state-space matrices are determined for the cantilever thick plate bonded with piezoelectric actuator and sensor pairs in optimal placement and optimal number based on Eq. (46). Then the optimal feedback control gain matrix calculates by using optimal linear quadratic control with weighted matrices ${\text{R}} = 1{}$ and ${\text{Q}} = 10^{5} ,\;10^{6} ,\;10^{7} ,\;10^{8}$. The closed-loop average vibration reduction using an optimal linear quadratic control scheme for the cantilever thick plate with ten sensor/ actuator pairs optimally located was compared with that of optimal piezoelectric configurations in the previously published study^18^,the result of the thin plate has been used on the thick plate), based on Eq. (43) and random piezoelectric configurations (Selection 10 numbers between 1 and 100 in randomly by MATLAB software), and the final results are reported in Table 4. It is clear that the present global optimal ten piezoelectric pairs (based on Eq. (46)) give an average improved vibration reduction between 17.49 and 40.95% compared to other sensor/actuator pairs configurations (see Table 5). Table 5 shows that percentage reduction improvement in the MCSVD approach is more than this value of the norm of SVD. This is also true for the random selection of piezoelectric configurations. In addition, using optimal locations for piezoelectric patches obtained on thin plates is not recommended for thick plates because there is less percentage reduction improvement than the value of using optimal placement based on thick plate (Eqs. (43) and (46)). An increase in the weighted matrix ${\text{Q}}$ to a value is higher than $10^{8}$ causes noise and locations divergence of the poles of the closed-loop system on the s-plane.

**Table 4:** Closed-loop average dB gain reduction for the cantilever thick bonded with ten piezoelectric sensor/actuator pairs.

Ten S/A pairs	Closed-loop dB gain reduction			
	${\text{Q}} = 10^{5}$	${\text{Q}} = 10^{6}$	${\text{Q}} = 10^{7}$	${\text{Q}} = 10^{8}$
Present study (Eq. (46))	11.22	13.02	15.12	18.00
Present study (Eq. (43))	8.87	10.53	12.56	15.32
Ref.^18^	8.06	9.82	11.84	14.67
Random	7.96	9.45	11.13	13.54

**Table 5:** Percentage reduction improvement for present study (Eq. (46)) than each case.

Ten S/A pairs	Percentage reduction improvement			
	${\text{Q}} = 10^{5}$	${\text{Q}} = 10^{6}$	${\text{Q}} = 10^{7}$	${\text{Q}} = 10^{8}$
Present study (Eq. (43))	26.49%	23.65%	20.38%	17.49%
Ref.^18^	39.21%	32.26%	27.70%	22.70%
Random	40.95%	37.78%	35.85%	32.94%

The systems related to the sensor-actuator pairs are introduced in Table 6. In this system, each actuator will be responsible to minimize only the sensor voltage right below it. Other techniques could be used aiming at the minimization of both sensor voltages simultaneously for each controller, but the results would be similar. The main reason for this result is that the control applied by each actuator affects its nearest sensor much more than the others.

**Table 6:** Optimal placement of piezoelectric pairs installed to the rectangular thick plate.

Controlled the first six modes	Number of patches									
	1	2	3	4	5	6	7	8	9	10
System number	System 1	System 2	System 3	System 4	System 5	System 6	System 7	System 8	System 9	System 10
Element number	23	28	29	35	41	48	54	60	73	92

**Figure 14:**
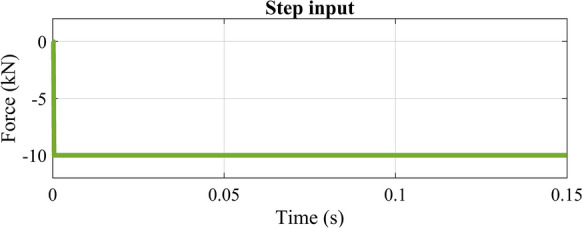
Step input force in ${\text{t}} = 0.0002{\text{s}}$ at (x, y, z) = (0, 0.269375 m, 0.03 m).

**Figure 15:**
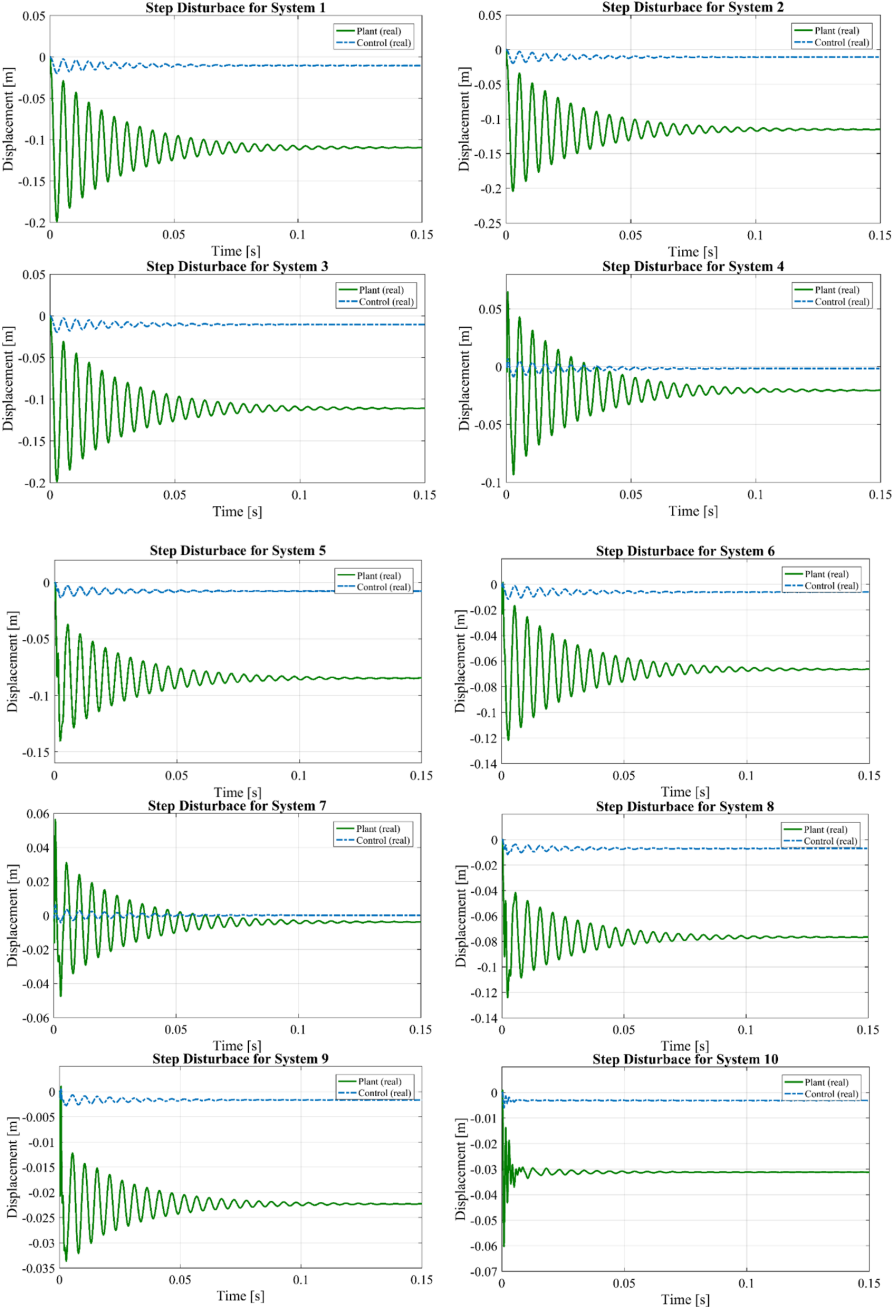
Step input: vertical displacement of the middle point with PID controller for all systems.

The vertical motion of the point where the force was applied (middle point) for step disturbance (Fig. 14) is represented in Fig. 15.

The maximum transverse displacement value for all systems in Fig. 15 is about 2 × 10^–1^ for impulse input. By using only 10 piezoelectric patches, the amplitude reduction is achieved over all 6 modes. The above results indicate that using an array of appropriately positioned discrete actuators is necessary to provide good controllability of the structure. The increased effectiveness is attributed to locating actuators in the best possible positions for control of all 6 structural modes. Therefore, the effect of optimal location of piezoelectric patches in suppressing displacement can be observed.

## Conclusions

In this paper, a binary-coded genetic algorithm which modified by adding feasibility test in the mutation step was applied to optimize the location and the number of piezoelectric patches on the cantilever thick plate in order to suppress the vibration. The objective function was obtained by modifying the control matrix and SVD (MCSVD). It is argued that the optimal positions and numbers calculated by the present method have raised the closed-loop average dB reduction gain compared with using random placement, norm of SVD, and the result of optimal placement on thin-plate. It has been found that the present optimal placement gives a higher average dB gain reduction of 26.49%, 23.65%, 20.38%, and 17.49% than the other cases at R = 1 and Q = 10^5^, 10^6^, 10^7^, 10^8^, respectively. The present objective function obtains a higher vibration reduction.

Future research in the respect of active vibration control includes number of topics dealing with the optimization of sensors and actuators, the type of structure considered and the theoretical and experimental application of different control schemes to suppress vibration. The following topics would be prolific areas for future investigation.

1. Investigation of active vibration reduction in stiffened plates, with and without symmetry, so as to implement the placement strategies and objective functions used in this study. Shells and plates stiffened by beams have been used intensively in the manufacturing of mechanical structures with high specific strength.
2. Investigation of reducing the search space in the optimization problem for active vibration control in flexible structures. A half-chromosome technique can be extended based on the finding that the global optimal configuration of actuators and sensors followed the structural dynamic axes of symmetry. The use of this technique greatly reduced the search space of the optimization problem and holds great potential for finding global optimal solution to large-scale mechanical structures after a small number of generations.
3. Further improvements to the effectiveness of active vibration control by optimal placement of sensors and actuators might be investigated using optimal proportional differential, LQR and fuzzy neural control schemes.

## Supplementary Information

Supplementary Information.
