# Supplementary material for: Optimal distribution of piezoelectric patches for active vibration reduction of a thick plate using singular value decomposition approach
Source: Sci Rep. 2021 Jul 2;11:13721. doi: 10.1038/s41598-021-93136-5 (PMC8253778; doi:10.1038/s41598-021-93136-5)
Supplement: Supplementary file 1 — Supplementary Information. [file 41598_2021_93136_MOESM1_ESM.pdf]

Supplementary Information for

# Optimal Distribution of Piezoelectric Patches for Active Vibration Reduction of a Thick Plate Using Singular Value Decomposition Approach.

Azin Nadi, Mojtaba Mahzoon, Ehsan Azadi Yazdi

**Supplementary Note: Thick plate formulation**

For a rectangular plate of width and thickness in the Cartesian coordinate system, the displacements and forces depend on the middle plane of the plate. Thick plate displacements include bending and shear displacements such as Tymoshenko's beam theory [1,2].

$$w(x,y,t) = w_b(x,y,t) + w_s(x,y,t) \tag{1}$$

where $w_b$ and $w_s$ are bending and shear displacement of thick plate, respectively.

The rotational angles of the cross-sectional area are related to the plate bending (Fig. 1).

$$\psi_x = \frac{\partial w_b}{\partial x}, \psi_y = \frac{\partial w_b}{\partial y} \tag{2}$$

The bending and torsion moments are related to the curvature of the plate (Fig. 2).

$$\begin{cases} M_x = -D\left(\frac{\partial^2 w_b}{\partial x^2} + \nu \frac{\partial^2 w_b}{\partial y^2}\right) \\ M_y = -D\left(\frac{\partial^2 w_b}{\partial y^2} + \nu \frac{\partial^2 w_b}{\partial x^2}\right) \\ M_{xy} = M_{yx} = -(1-\nu)D\frac{\partial^2 w_b}{\partial x \partial y} \end{cases} \tag{3}$$

Transverse shear forces

$$Q_x = S\frac{\partial w_s}{\partial x}, Q_y = S\frac{\partial w_s}{\partial y} \tag{4}$$

In the Eq. (3) and (4), $D = \frac{Eh^3}{12(1-\upsilon^2)}$ and $S = kGh$ are the bending and shear strength of the plate, respectively. $E, G, \upsilon, k$ and $h$ are Young's modulus, Shear modulus, Poisson's ratio, shear correction coefficient and plate thickness, respectively.

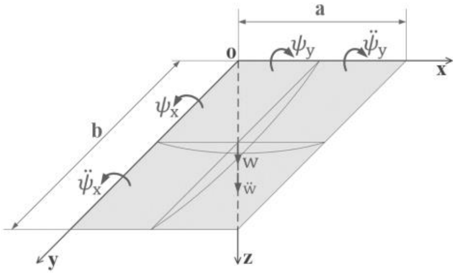


**Supplementary Figure 1.** Rectangular displacements.

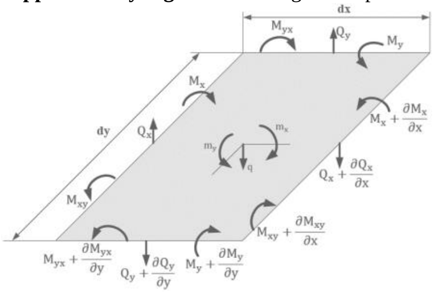


**Supplementary Figure 2.** Internal and External Forces on the Differential Element Plate.

$$q = -\bar{m}\frac{\partial^2 w}{\partial t^2}, m_x = -J\frac{\partial^3 w_b}{\partial x \partial t^2}, m_y = -J\frac{\partial^3 w_b}{\partial y \partial t^2} \tag{5}$$

where $\bar{m} = \rho h$ is the mass per the unit area, $\rho$ is density of the plate, and $J = \rho I = \rho(\frac{h^3}{12})$ is mass inertia moment of plate cross-section.

**Vibrational differential equations**

The differential element of the plate represents the bending and torsional moments, shear forces and inertial loads. The equilibrium equation of $x$ and $y$ axes and forces is written in the line $z$.

$$\begin{cases} \frac{\partial M_x}{\partial x} + \frac{\partial M_{xy}}{\partial y} = Q_x + m_x \\ \frac{\partial M_y}{\partial y} + \frac{\partial M_{yx}}{\partial x} = Q_y + m_y \\ \frac{\partial Q_x}{\partial x} + \frac{\partial Q_y}{\partial y} = -q \end{cases} \tag{6}$$

By substituting (3), (4) and (5) into (6), it is obtained that.

$$\begin{cases} \frac{\partial w_s}{\partial x} = -\frac{D}{S}(\frac{\partial^3 w_b}{\partial x^3} + \frac{\partial^3 w_b}{\partial x \partial y^2}) + \frac{J}{S}\frac{\partial^3 w_b}{\partial x \partial t^2} \\ \frac{\partial w_s}{\partial y} = -\frac{D}{S}(\frac{\partial^3 w_b}{\partial y^3} + \frac{\partial^3 w_b}{\partial x^2 \partial y}) + \frac{J}{S}\frac{\partial^3 w_b}{\partial y \partial t^2} \\ \frac{\partial^2 w_s}{\partial x^2} + \frac{\partial^2 w_s}{\partial y^2} - \frac{\bar{m}}{S}\frac{\partial^2 w_b}{\partial t^2} - \frac{\bar{m}}{S}\frac{\partial^2 w_s}{\partial t^2} = 0 \end{cases} \quad (7)$$

By integrating the first two equations of the Eq. (7) in the order of the variables $x$ and $y$

$$\begin{cases} w_s = -\frac{D}{S}\left(\frac{\partial^2 w_b}{\partial x^2} + \frac{\partial^2 w_b}{\partial y^2}\right) + \frac{J}{S}\frac{\partial^2 w_b}{\partial t^2} + f_y(y,t) \\ w_s = -\frac{D}{S}\left(\frac{\partial^2 w_b}{\partial y^2} + \frac{\partial^2 w_b}{\partial x^2}\right) + \frac{J}{S}\frac{\partial^2 w_b}{\partial t^2} + f_x(x,t) \end{cases} \quad (8)$$

By equating two relationships (8), $f_y(y,t) = f_x(x,t) = f(t)$ Finally, the relation (8) is rewritten as follows.

$$w_s = -\frac{D}{S}\nabla^2 w_b + \frac{J}{S}\frac{\partial^2 w_b}{\partial t^2} + f(t) \quad (9)$$

where $\nabla^2(.) = \frac{\partial^2(.)}{\partial x^2} + \frac{\partial^2(.)}{\partial y^2}$ is the Laplace differential operator.

The function $f(t)$ represents the movement of the rigid body and can be ignored. By placing (9) in the last relation of Eq. (7) a vibrational differential equation is obtained only for the term $w_b$.

$$D\nabla^4 w_b - J\left(1 + \frac{\bar{m}D}{JS}\right)\frac{\partial^2}{\partial t^2}\nabla^2 w_b + \bar{m}\frac{\partial^2}{\partial t^2}\left(w_b + \frac{J}{S}\frac{\partial^2 w_b}{\partial t^2}\right) = 0 \quad (10)$$

where $\nabla^4$ is a differential oprator equivalent to $\frac{\partial^4}{\partial x^4} + 2\frac{\partial^4}{\partial x^2 \partial y^2} + \frac{\partial^4}{\partial y^4}$.

## Supplementary References


1. O. Abdeljaber, O. Avci, D.J. Inman, Active vibration control of flexible cantilever plates using piezoelectric materials and artificial neural networks, *J. Sound Vib.* **363**, 33–53 (2015).
2. I. Senjanović, N. Vladimir, M. Tomić, An advanced theory of moderately thick plate vibrations, *J. Sound Vib.* **332**, 1868–1880 (2013).
